# Exacerbated inflammatory signaling underlies aberrant response to BMP9 in pulmonary arterial hypertension lung endothelial cells

**DOI:** 10.1007/s10456-020-09741-x

**Published:** 2020-08-19

**Authors:** Robert Szulcek, Gonzalo Sanchez-Duffhues, Nina Rol, Xiaoke Pan, Roula Tsonaka, Chris Dickhoff, Lai Ming Yung, Xue D. Manz, Kondababu Kurakula, Szymon M. Kiełbasa, Hailiang Mei, Wim Timens, Paul B. Yu, Harm-Jan Bogaard, Marie-José Goumans

**Affiliations:** 1 Department of Cell and Chemical Biology, Leiden University Medical Center (LUMC), Leiden, The Netherlands; 2 Department of Pulmonary Diseases, Amsterdam UMC, VU University Medical Center (VUmc), Amsterdam Cardiovascular Sciences (ACS), Amsterdam, The Netherlands; 3 Department of Biomedical Data Sciences, Medical Statistics Section, LUMC, Leiden, The Netherlands; 4 Department of Surgery, VUmc, Amsterdam, The Netherlands; 5 Cardiovascular Division, Brigham and Women’s Hospital, Harvard Medical School, Boston, MA USA; 6 Sequencing Analysis Support Core, LUMC, Leiden, The Netherlands; 7 Department of Pathology and Medical Biology, Universtiy of Groningen, University Medical Center Groningen, Groningen, The Netherlands

**Keywords:** Pulmonary hypertension, Pulmonary endothelial cells, Endothelial-to-mesenchymal transition, Bone morphogenetic protein, Interleukin-6

## Abstract

Imbalanced transforming growth factor beta (TGFβ) and bone morphogenetic protein (BMP) signaling are postulated to favor a pathological pulmonary endothelial cell (EC) phenotype in pulmonary arterial hypertension (PAH). BMP9 is shown to reinstate BMP receptor type-II (BMPR2) levels and thereby mitigate hemodynamic and vascular abnormalities in several animal models of pulmonary hypertension (PH). Yet, responses of the pulmonary endothelium of PAH patients to BMP9 are unknown. Therefore, we treated primary PAH patient-derived and healthy pulmonary ECs with BMP9 and observed that stimulation induces transient transcriptional signaling associated with the process of endothelial-to-mesenchymal transition (EndMT). However, solely PAH pulmonary ECs showed signs of a mesenchymal trans-differentiation characterized by a loss of VE-cadherin, induction of transgelin (SM22α), and reorganization of the cytoskeleton. In the PAH cells, a prolonged EndMT signaling was found accompanied by sustained elevation of pro-inflammatory, pro-hypoxic, and pro-apoptotic signaling. Herein we identified interleukin-6 (IL6)-dependent signaling to be the central mediator required for the BMP9-induced phenotypic change in PAH pulmonary ECs. Furthermore, we were able to target the BMP9-induced EndMT process by an IL6 capturing antibody that normalized autocrine IL6 levels, prevented mesenchymal transformation, and maintained a functional EC phenotype in PAH pulmonary ECs. In conclusion, our results show that the BMP9-induced aberrant EndMT in PAH pulmonary ECs is dependent on exacerbated pro-inflammatory signaling mediated through IL6.

## Introduction

Progressive occlusive remodeling of the distal pulmonary vasculature is the hallmark of pulmonary arterial hypertension (PAH), a heterogenous group of deadly lung disorders clinically defined by a highly increased mean pulmonary artery pressure at rest in the absence of other causes of pre-capillary pulmonary hypertension (PH) [1, 2]. Histologically, PAH is associated with a dramatic reorganization of the pulmonary arterial architecture involving medial as well as intimal thickening, an extensive loss of capillaries, and the appearance of characteristic, disorganized plexogenic lesions that are highly enriched with alpha-smooth muscle actin (α-SMA) positive cells [3]. Phenotypically altered, de-differentiated, partially dysfunctional pulmonary endothelial cells (ECs) are postulated to contribute to the occlusive vascular remodeling in PAH both directly by transforming into smooth muscle (SM)-like cells as well as indirectly through paracrine effects [4–6]. Endothelial-to-mesenchymal transition (EndMT) is an essential developmental process by which mature ECs lose their specific protein expression, morphology, and polarity to acquire mesenchymal characteristics and has moved into focus as a possible source of these highly proliferative SM-like mesenchymal cells in PAH [7]. Notably, induction of EndMT requires the coordinated action of multiple signaling cascades, induced by both circulating factors and tissue-specific stimuli [8].

The transforming growth factor beta (TGFβ) family contains central drivers and modulators of the EndMT process and is essential in the control of the EC phenotype [8]. Interestingly, imbalanced TGFβ signaling is a characteristic feature in all PAH subtypes that includes loss-of-function genetic mutations in components of the bone morphogenetic protein (BMP) signaling pathway (*i.e., ACVRL1*, *BMPR1B*, *BMPR2*) and reduced expression of the BMP type-II receptor (BMPR2) in mutation-positive and -negative cases of PAH [9]. This shift is associated with decreased BMP-dependent signaling and increased TGFβ-responsiveness in pulmonary ECs of PAH patients [10]. Consequently, novel experimental treatment efforts aim to restore BMPR2 levels and consecutive downstream signaling to reinstate the balance in TGFβ/BMP activity [11], for example, by administration of BMP ligands or agonists [12–14].

Although initially discovered by their ability to induce ectopic bone formation in rodents [15], BMPs have been unveiled as pleiotropic molecules, which play a central role in cell differentiation, organogenesis, vascular development, and vascular homeostasis [16]. Particularly in ECs, BMP9, the ALK1 receptor high affinity ligand [17], is generally described as a circulating vascular quiescence and maintenance factor that can exert hematopoietic, hepato-, osteo-, chondro-, and adipogenic functions in a highly context and concentration-dependent manner [18]. In the mature endothelium, BMP9 appears to have anti-angiogenic and anti-apoptotic effects [14, 18] and is a known inducer of EndMT during embryonic development and thereby, for instance, controls vascular remodeling and vascular wall-thickening [7, 19]. However, BMP9 also serves as a pro-angiogenic and pro-tumorigenic factor in cancer cells demonstrating its pleiotropic roles in health and disease [20].

In the context of PAH, recombinant BMP9 administration has been shown to induce BMPR2 expression in blood-derived circulating ECs from PAH patients carrying different heterozygous *BMPR2* mutations and has beneficial hemodynamic and anti-remodeling effects in PH animal surrogate models when applied preventively or therapeutically [14]. However, the effects of BMPs on the primary endothelium are highly context-dependent [21]. The concern that the same ligand might have opposite effects is illustrated by contradicting reports showing that genetic deletion or pharmacological inactivation of BMP9 protects rodents from experimental PH [22], while a case study associates a homozygous nonsense mutation in *GDF2* (encoding for BMP9) with the development of PAH in infants [23], and *BMPR2* loss-of-function mutations are known to alter the tissue microenvironment [24].

To gain insights into the effect of BMP9 on the pulmonary vascular endothelium, in this manuscript, we comprehensively examine effects of BMP9 on control and PAH primary pulmonary EC signaling and phenotype. We identify a novel mechanism modulated through interleukin-6 (IL6) by which BMP9 triggers EndMT in PAH pulmonary ECs. Our discovery of the cell phenotype modulating function by combined action of IL6 and BMP9 will contribute to the understanding of the pathological mechanisms driving PAH-specific vascular changes and may eventually aid in the development of a treatment for this currently untreatable disease.

## Material and methods

### Cell cultures and in-vitro assays

The institutional review board (IRB) for human studies of the VU University Medical Center (Amsterdam, the Netherlands) approved the study protocols (non-WMO, 2012/306) and written informed consent was obtained from the subjects or their surrogates for the collection of materials and publication of results, if required. Microvascular ECs were isolated from pleura-free peripheral lung tissues, pulmonary artery ECs from rings of the *arteria pulmonalis*, and circulating ECs from heparinized peripheral blood, as described previously [25, 26]. Human PAH lung tissues were obtained from end-stage patients undergoing lung transplantations or from autopsies. Control tissues from lobectomies for suspected or proven non-small cell lung cancer (NSCLC) without PH were assessed by a pathologist and only normal tissues were used for cell isolations. Donor characteristics can be found in Table 1.

**Table 1:** Patient characteristics

ID	Assays	Diagnosis	FVC	FEV1	Dyspnea	Sex	Age	Ethnicity	Source	Echo/CT
MVECs used in the control group										
Ctrl01	PCR, RNA-seq, IF, ELISA, ECIS	NSCLC, adenocarcinoma	–	–	No	F	55	Caucasian	Lob	No dilation of RV, RA, or LV
Ctrl02	PCR, RNA-seq, IF, ELISA	NSCLC, squamous cell carcinoma	3.11 (100%)	2.31 (98%)	No	M	79	Caucasian	Lob	No dilation of RV, RA, or LV
Ctrl03	PCR, RNA-seq, IF, ECIS	NSCLC	5.3 (96%)	4.39 (98%)	No	M	42	Caucasian	Lob	No dilation of RV, RA, or LV
Ctrl04	IF, ELISA, ECIS	NSCLC	4.13 (110%)	3.23 (110%)	No	F	60	Caucasian	Lob	No dilation of RV, RA, or LV
Ctrl05	PCR, RNA-seq, ECIS	NSCLC, squamous cell carcinoma	2.75 (100%)	1.17 (50%)	No	F	61	Caucasian	Lob	No dilation of RV, RA, or LV
Ctrl06	PCR, RNA-seq	Tumoral obstruction	–	–	Yes	M	42	Caucasian	Lob	Enlarged RV, small LV, enlarged RA

ID	Assays	Diagnosis	mPAP	PVR	CI	Sex	Age	Ethnicity	Source	Treatment
MVECs used in the PAH group										
PAH01	PCR, RNA-seq, ELISA	iPAH	54	–	2.1	F	54	Caucasian	Obd	PDE5-I, ERA, PGI2
PAH02	PCR, RNA-seq, IF, ELISA, ECIS	hPAH (*BMPR2*)	68	–	1.6	F	40	Caucasian	Ltx	PDE5-I, ERA, PGI2
PAH03	PCR, RNA-seq, ELISA, ECIS	iPAH	43	620	2.1	F	42	Caucasian	Ltx	PDE5-I, PGI2
PAH04	PCR, RNA-seq, IF, ELISA, ECIS	iPAH	89	1527	1.9	F	22	Caucasian	Ltx	PDE5-I, ERA, PGI2
PAH05	PCR, RNA-seq, IF, ELISA, ECIS	iPAH	102	1375	3.4	M	21	Caucasian	Ltx	PDE5-I, ERA, PGI2

*ECIS* barrier function, *IF* immunofluorescence, *MVECs *lung microvascular endothelial cells, *NSCLC* non-small-cell lung carcinoma, *FVC* forced vital capacity (*L*), *FEV1* first second of forced expiration (*L*), *Lob* lobectomy, *RV* right ventricle, *RA* right atrium, *LV* left ventricle, *iPAH* idiopathic pulmonary arterial hypertension, *hPAH* hereditary PAH, *mPAP* mean pulmonary artery pressure (mmHg), *PVR* pulmonary vascular resistance (WU), *CI* cardiac index (L/min/m^2^), *PDE5-I* phosphodiesterase type 5 inhibitor, *PGI2* prostacyclin, *ERA* endothelin receptor antagonist, *Obd* autopsy, *Ltx* lung transplantation. PAH patients hemodynamics were determined by right heart catheterization before lung transplantation

ECs were purified by magnetic affinity cell sorting (MACS, Miltenyi Biotec) based on CD144 (VE-cadherin) antibody labeling and purity was ensured by regular FACS testing. Cells were cultured on 0.1% gelatin-coated standard cultureware (Corning) in ECM medium supplemented with 1% pen/strep, 1% endothelial cell growth supplement, 5% FCS (all ScienCell), and 1% non-essential amino acids (Biowest). Treatments were performed after 5 h preparative serum starvation with 1% FCS and without additional growth factors. Stimuli were made fresh in final concentrations of 1 ng/mL BMP9 (R&D Systems), 1 ng/mL TGFβ1 (Sigma), 10 ng/mL IL6 (BD Biosciences), and 10 ng/mL IL6 blocking antibody (mabg-hil6-3, InvivoGen).

Barrier function was determined by impedance spectroscopy with ECIS (Electric Cell-substrate Impedance Sensing, Applied Biophysics). Resistance was analyzed and modeling of cell–cell and cell–matrix strength carried out as described previously [27]. ELISAs for IL6 on cell-free supernatants were carried out with the BD OptEIA human IL6 kit (BD Bioscience) and on human serum with the IL6 kit from Antigenix following the manufacturer’s instructions.

### Real-time polymerase chain reaction (RT-PCR)

RNA was isolated with the miRNeasy mini kit (Qiagen), cDNA synthesis performed with the iScript cDNA synthesis kit (Bio-Rad) on a 2720 Thermal Cycler (Applied BioSystems), and RT-PCR carried out with iQ SYBR green supermix on a CFX384 Real-Time System (all Bio-Rad) following the manufacturer’s instructions. Primer details (Sigma-Aldrich) can be found in Table 2.

**Table 2:** Primer list for human genes

Seq ID	Name	Sequence 5′–3′
NM_001204.6	*BMPR2_Fwd*	*GTCCTGGATGGCAGCAGTAT*
	*BMPR2_Rev*	*CCAGCGATTCAGTGGAGATGA*
NM_002165.3	*ID1_Fwd*	*CTGCTCTACGACATGAACGG*
	*ID1_Rev*	*GAAGGTCCCTGATGTAGTCGAT*
NM_002167.4	*ID3_Fwd*	*CACCTCCAGAACGCAGGTGCTG*
	*ID3_Rev*	*AGGGCGAAGTTGGGGCCCAT*
NM_000602.4	*PAI1_Fwd*	*CAATCGCAAGGCACCTCTGA*
	*PAI1_Rev*	*TTCACCAAAGACAAGGGCCA*
NM_005985.3	*SNAI1_Fwd*	*ACCACTATGCCGCGCTCTT*
	*SNAI1_Rev*	*GGTCGTAGGGCTGCTGGAA*
NM_003068.4	*SNAI2_Fwd*	*TCGGACCCACACATTACCTT*
	*SNA2_Rev*	*TGAGCCCTCAGATTTGACCT*
NM_000600.4	*IL6_Fwd*	*ACAGCCACTCACCTCTTCAG*
	*IL6_Rev*	*GCAAGTCTCCTCATTGAATCCAG*
NM_001002.3 House Keeping Gene	*P0_Fwd*	*TCGACAATGGCAGCATCTAC*
	*P0_Rev*	*ATCCGTCTCCACAGACAAGG*
NM_001289746.1 House Keeping Gene	*GAPDH_Fwd*	*GGTCTCCTCTGACTTCAACA*
	*GAPDH_Rev*	*AGCCAAATTCGTTGTCATAC*
NR_146119.1; NR_145820.1 House Keeping Gene	*18S_Fwd*	*AACGGCTACCACATCCAAGG*
	*18S_Rev*	CAGCTAAGAGCATCGAGGGG

### Western blot

Gel electrophoresis was run with NuPAGE 4–12% Bis–Tris pre-cast gels and the accompanying buffers (Invitrogen) following the manufacturer’s instructions. Antibodies against BMPR2 (1:2000, Ma5-15827, Thermo Fisher Scientific), pSMAD1/5/9 (1:1000, 13820, Cell Signaling), pSMAD2 (1:1000, gift from Prof. ten Dijke at LUMC Leiden), and GAPDH (1:10000, g9295, Sigma-Aldrich) were used for protein detection.

### Global transcriptomics (RNA-seq) and analysis

Serum-starved microvascular lung ECs (5 h at 1% FCS, no growth factors) were either stimulated with BMP9 (for 90 min or 24 h) or left untreated. RNA was isolated with the miRNAeasy mini kit (Quiagen). Total RNA was purified using MagMAX-96 total RNA isolation kit (Ambion), in which genomic DNA was removed. mRNA was purified from total RNA using Dynabeads mRNA purification kit (Invitrogen). Strand-specific RNA sequencing libraries were prepared using ScriptSeq mRNA-seq library preparation kit (Epicenter). Sequencing was performed on HiSeq2000 (Illumina) by a multiplexed, single-read run with 33 cycles. Reads were mapped to the human genome hg38. Differential gene expression analysis was performed by the Medical Statistics and Bioinformatics core at LUMC using normalized log-transformed counts per gene with appropriate weights per observation in a fdr multiple testing corrected multivariate regression model. The model tested which genes are differentially expressed between the three conditions (starved, 90-min, or 24-h stimulation) in at least one donor group (control *vs.* PAH). Gene Set Enrichment Analysis (GSEA) was run with the pre-ranked tool [28] on the adjusted log2-fold gene lists. Pathway enrichment was defined by FDR < 0.05 and *p* < 0.001. Enrichment map visualization (network graph) was done with the Enrichment Map Pipeline collection in Cytoscape version 3.6.1.

### Immunofluorescence staining

Human ECs were fixed in warm 4% paraformaldehyde for 20 min at room temperature (RT), quenched with 2 mg/mL glycine, permeabilized with 0.2% Triton X-100 for 10 min at RT, blocked with 5% BSA, and labeled with VE-cadherin (1:500, 2158, Cell Signaling), SM22α (1:500, ab14106, Abcam)-specific antibodies, and/or Rhodamine-Phalloidin (1:1000, R415, Invitrogen). Samples were preserved in ProLong Gold anti-fading agent with DAPI (Thermo Fisher Scientific). Imaging was done on a Nikon A1 confocal laser microscope at ×60 magnification. Image quantification was performed with ImageJ (NIH) by measuring VE-cadherin and SM22α intensity of a total of nine individual cells per donor at three random locations in the culture well. The resulting intensity values were normalized to the mean intensity of the unstimulated controls within one experiment. F-actin orientation was analyzed using the directionality function in ImageJ on images from three random locations in the culture.

### Statistics

Individual cell culture experiments were repeated at least three times, with different combinations of available donors. Numbers of used donors are indicated within figures or by *n* in figure legends. Experimental data were analyzed by Student’s *t* tests, multiple corrected *t* tests, and one-way or two-way ANOVAs where applicable. The appropriate statistical tests are specified in the figure legends. Data were considered significantly different at *p* values ≤ 0.05. Data were visualized using GraphPad Prism version 7 (GraphPad Software). If not indicated differently, data are presented as mean ± standard deviation.

## Results

### PAH pulmonary endothelial cells respond different to BMP9

**Fig. 1:**
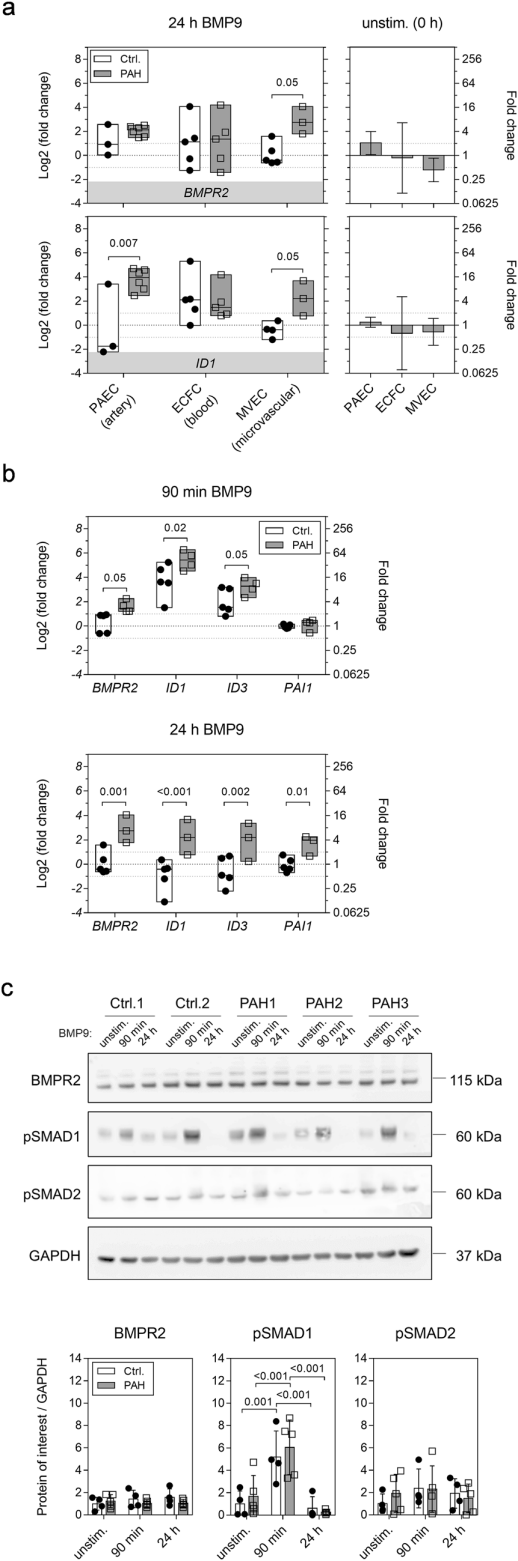
The human microvascular lung endothelium of PAH patients shows a distinct transcriptional response to long-term BMP9 stimulation. **a** Box plots show RT-PCR analysis of gene expression in lung microvascular (MVECs), pulmonary arterial (PAECs), and peripheral blood-derived circulating (ECFCs) endothelial cells in response to 24-h activation with BMP9. Data are presented as min, max, and median log2-fold changes compared to unstimulated conditions. Bar graphs represent mean basal gene expression with appropriate confidence intervals before BMP9 addition in PAH samples compared to controls. **b** RT-PCR quantification of gene regulation in MVECs after 90-min or 24-h BMP9 compared to unstimulated levels. **c** Representative Western blot from whole MVEC lysates. Bar graphs contain protein quantifications of all tested donors normalized to GAPDH. Two-way ANOVAs with Tukey multiple comparison correction were applied to calculate *p* values in between donor groups (ctrl. vs. PAH, **a** and **b**) or all individual conditions and donors (**c**)

Smaller pulmonary arteries that contain highly specialized microvascular endothelial cells (MVECs) are the principal sites of vascular remodeling in PAH [3]. However, peripheral blood-derived endothelial colony-forming cells (ECFCs) are often used as surrogates for pulmonary ECs [14]. We compared BMP9 responses between ECFCs, MVECs, and pulmonary artery endothelial cells (PAECs) to get an indication of potential endothelial subtype-specific differences (Fig. 1a). Transcriptional activation of *BMPR2* and the BMP target gene *ID1* were used as read-outs for BMP pathway activation. Basal gene expression was not different between control and PAH cells. ECFCs reacted to 24-h BMP9 stimulation with a median two-fold increase in *BMPR2* and approximately four-fold increase in *ID1* gene expression that was similar between the two donor groups (PAH *vs*. controls). PAH patient-derived PAECs responded to BMP9 with a four-fold, respectively, 16-fold upregulation of the two tested genes. In PAH PAECs, *ID1* was significantly different from controls (*p* = 0.007), with controls even trending towards decreased expression upon BMP9. MVECs from PAH patients responded differently to BMP9 than PAECs and ECFCs showing a significant eight-fold increase in *BMPR2* compared to unaltered controls (*p* = 0.05). Similarly, *ID1* increased by four-fold in PAH MVECs (*p* = 0.05), whereas controls expressed basal levels.

Possible time-dependent effects in lung MVECs were explored by determining transcriptional regulation in response to short- (90-min) or long-term (24-h) BMP9 stimulation (Fig. 1b). Short-term stimulation induced a robust activation of the BMP pathway shown by increased *ID1* and *ID3* expression, while the TGFβ-target *PAI1,* an indicator of non-canonical effects of BMP9, remained at levels of unstimulated samples. Interestingly, *BMPR2* significantly increased three-fold (*p* = 0.05) in PAH cells, whereas controls remained at basal levels. Pronounced differences were seen between control and PAH cells at the 24-h time point. BMP9 elicited four-fold induction of all tested genes in PAH cells indicating a sustained activation of BMP and TGFβ transcriptional targets, whereas in controls, levels were like unstimulated suggesting an altered response to BMP9 in PAH MVECs.

On protein level, short-term BMP9 treatment induced a significant activation of BMP-dependent signaling by phosphorylation of SMAD1 (*p* ≤ 0.001) that was similar in controls and PAH MVECs (Fig. 1c). BMPR2 and phospho-SMAD2 protein levels did not change upon BMP9 stimulation and no differences between the PAH MVECs and controls were found. No BMP9-dependent protein response was detectable after 24 h.

In summary, the response of ECFCs to BMP9 was highly variable between donors and not different between control and PAH samples. On the contrary, pulmonary MVECs and PAECs from PAH patients displayed dysregulated gene activation following BMP9 stimulation that was most pronounced in the microvascular endothelium after 24 h. We performed comprehensive transcriptional analysis in PAH MVECs to gain a deeper understanding of the mechanisms underlying this altered response to BMP9.

### BMP9 is a potent inducer of EndMT transcriptional signaling

**Fig. 2:**
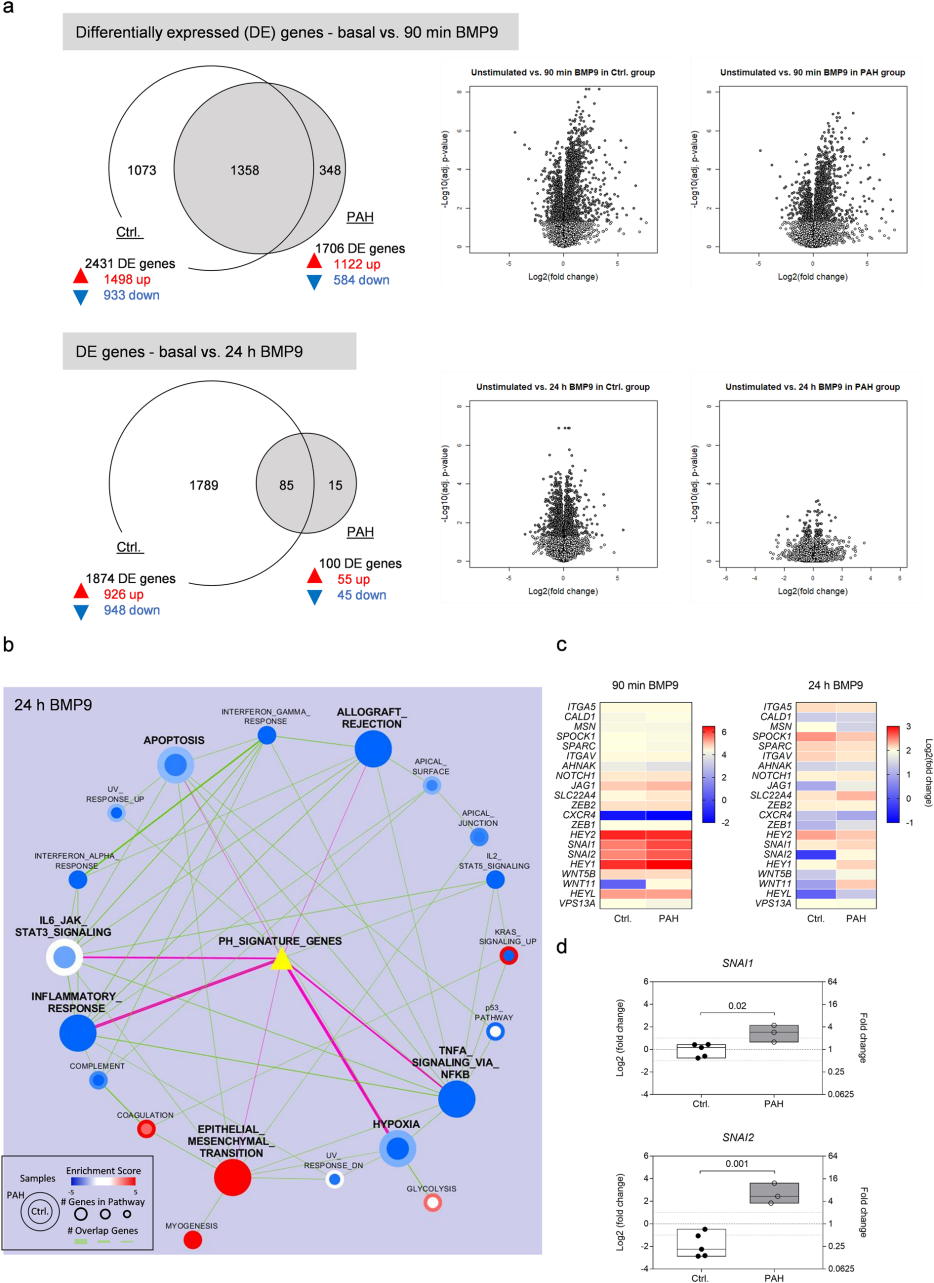
Long-term homeostatic responses to BMP9 are disturbed in the PAH lung endothelium. **a** Global transcriptome analysis (RNA-seq, *n* = 5) on MVECs receiving BMP9 for 90 min or 24 h. Differential gene expression was calculated in comparison to unstimulated samples. Venn diagrams illustrate the number of overlapping genes that pass the log2-fold threshold of ± 1 within control or PAH groups. Volcano plots visualize all genes passing the *p* value threshold of *p* ≤ 0.05 (dark grey dots). **b** Network graph resulting from unbiased Gene Set Enrichment Analysis (GSEA) in control (inner circle) and PAH MVECs (outer circle) at 24 h after BMP9 stimulation. Pathway activation was compared to unstimulated samples. Represented are positive (up-regulated, red circles), negative (downregulated, blue circles), and non-enriched pathways (white circles); gene overlap between pathways (green lines); and gene overlap with a pulmonary hypertension (PH) signature gene set (yellow triangle with magenta lines, overlap cut-off ≥ 5 genes). Thickness of lines indicates number of overlapping genes. **c** Heat maps of genes induced in endothelial-to-mesenchymal transition (EndMT). Shown are log2-fold changes in transcript levels following 90-min or 24-h BMP9 stimulation compared to basal samples. **d** RT-PCR validation of the EndMT master transcription factors *SNAI1* and *SNAI2*. Box plots represent min, max, and median log2-fold changes after 24-h BMP9 compared to unstimulated conditions. Unpaired Student’s *t* tests were used to calculate *p* values

RNA sequencing was carried out on the short- and long-term BMP9-stimulated MVECs, since the sustained elevated levels of *BMPR2, ID1/3,* and *PAI1* might indicate dysfunctional negative feedback signaling in PAH cells. The transcriptome of controls and PAH samples overlapped substantially after 90 min of BMP9 stimulation with 55.9% of all control genes that passed the log2-threshold of ± 1 intersecting with the differentially expressed genes (DE) in the PAH group (Fig. 2a). The number of intersecting DE genes decreased to 4.5% at 24 h, again pointing towards altered long-term homeostatic responses in PAH pulmonary ECs.

Unbiased Gene Set Enrichment Analysis (GSEA) [28] was performed to get an overview on signaling in response to long-term BMP9 stimulation. Active pathways and their regulation are illustrated in the GSEA pathway enrichment map (Fig. 2b). To highlight pathways associated with the pathogenesis of the disease and to perform targeted analysis, the STRING database [29] was queried for “Pulmonary Hypertension". The resulting gene list that included 100 genes was overlaid onto the network map (cut-off ≥ 5 overlapping genes). BMP9 caused downregulation of almost all active pathways at 24 h except for epithelial-to-mesenchymal transition (EMT) signaling, which showed a robust positive enrichment (upregulation) in both control and PAH samples.

Next, a previously published EMT/EndMT signature gene panel [30] was applied to the analysis to determine directionality of pathway-associated gene regulation (Fig. 2c). A gene cluster comprising the EndMT-associated transcription factors *SNAI1*, *SNAI2*, *HEY1,* and *HEY2* was found three to six log2-fold upregulated in both control and PAH MVECs at 90 min compared to unstimulated conditions. After 24 h, *HEY1*/*2* and *SNAI1* showed decreased but still comparable levels in controls *vs*. PAH. On the contrary, *SNAI2* was found −1 log2-fold downregulated in controls while PAH samples still expressed two log2-fold increased levels compared to unstimulated conditions.

RT-PCR validation was carried out for *SNAI1* and *SNAI2* regulation upon long-term BMP9 stimulation (Fig. 2d). The PCR corroborated the transcriptomic analysis of a robust elevation in *SNAI1* (1.52 ± 0.75 log2(FC), *p* = 0.02) and especially *SNAI2* expression (2.44 ± 0.89 log2(FC), *p* = 0.001) in PAH cells compared to unstimulated samples. Controls, on the contrary, expressed basal values of *SNAI1* (0.17 ± 0.57 log2(FC)) and even downregulated *SNAI2* (− 2.25 ± 1.07 log2(FC)).

### Long-term treatment with BMP9 induces EndMT in PAH MVECs

**Fig. 3:**
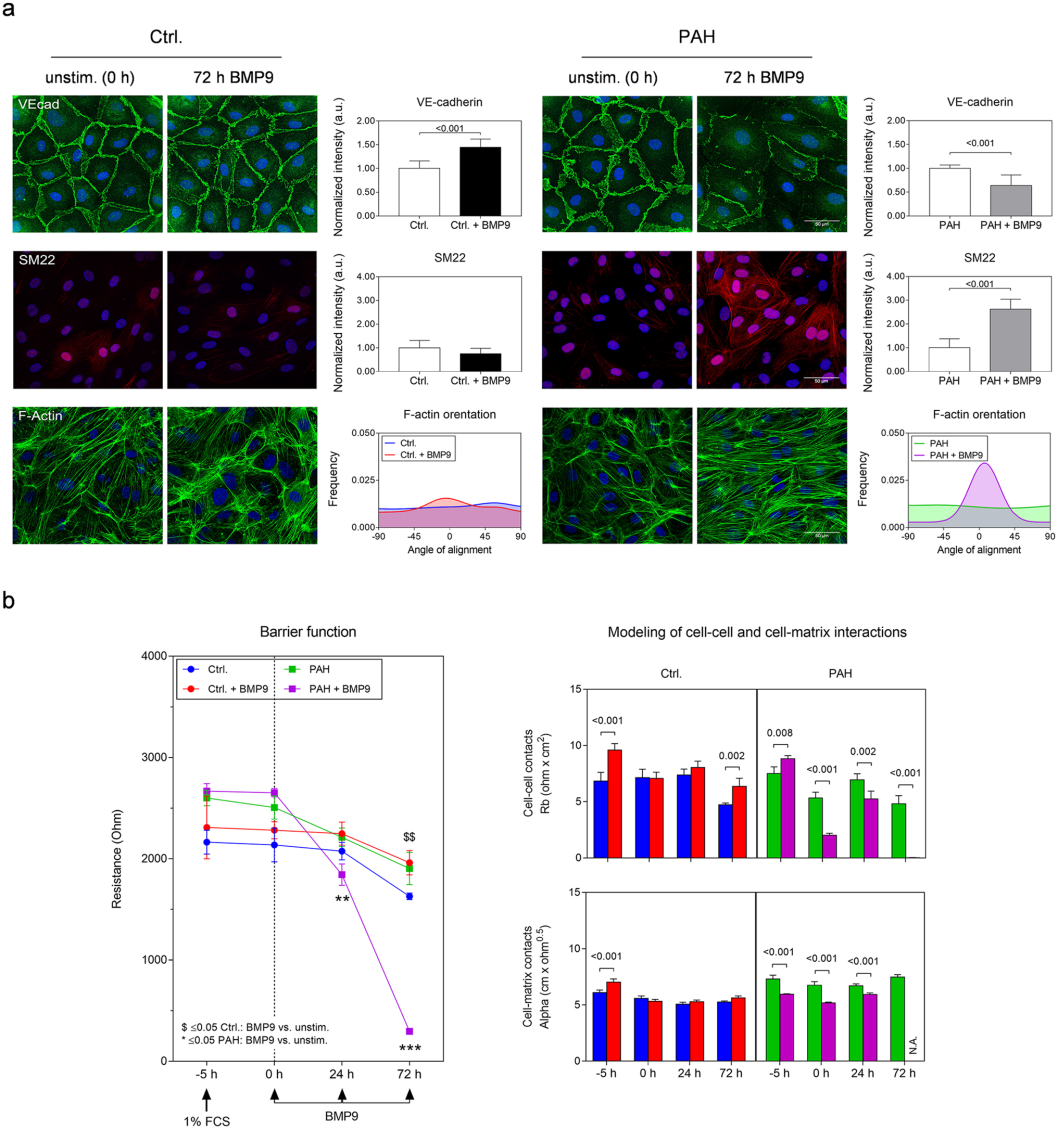
Repetitive, long-term BMP9 stimulation pushes the PAH lung microvascular endothelium into a mesenchymal phenotype. **a** Representative staining of confluent MVECs for the endothelial marker VE-cadherin, the mesenchymal marker transgelin (SM22α), and the cytoskeletal protein F-actin after repetitive BMP9 stimulation every 24 h for a total duration of three days. Bar graphs represent quantifications (*n* = 3) of VE-cadherin and SM22 intensity as well as F-actin fiber orientation. Statistical differences were determined by unpaired *t* tests. **b** Time-resolved impedance spectroscopic quantification of endothelial barrier function (resistance, *n* = 4). BMP9 was administered after 5 h preparative serum starvation with 1% FCS every 24 h for a total duration of three days (arrow heads). Bar graphs represent integrity and strength of cell–cell (Rb) and cell–matrix interactions (Alpha) mathematically modeled from the impedance data. N.A. indicates inability to model data because of too low impedance values. Multiple *t* tests with Holm–Sidak corrections were used to calculate statistics for resistance within the control or the respective PAH group. Two-way ANOVA with Tukey multiple comparison correction was used to calculate differences in adhesion strength between ctrl. and PAH samples at the individual time points

Based on the transcriptomic findings, we tested the impact of BMP9 on phenotypic plasticity in PAH pulmonary ECs. Human MVECs received daily BMP9 supplementation for a total duration of three days and were fluorescently labeled for endothelial and mesenchymal markers (Fig. 3a). Control cells significantly increased peripheral expression of endothelial VE-cadherin in response to BMP9 (1.00 ± 0.16 *vs*. 1.45 ± 0.17, *p* < 0.001), retained low levels of SM22α, and maintained a characteristic EC cobble stone morphology with well-organized peripheral F-actin. PAH patient-derived MVECs effectively lost VE-cadherin expression from cell–cell junctions (1.00 ± 0.07 *vs*. 0.64 ± 0.22, *p* < 0.001) and displayed an elongated morphology with F-actin stress fibers gaining collective directionality and spanning the entire cell body. In a subset of the stimulated PAH cells, SM22α expression increased significantly (1.00 ± 0.38 *vs*. 2.62 ± 0.42, *p* < 0.001) and organized in a mesenchymal-like pattern.

Disruption of endothelial junctions due to EndMT may contribute to PAH pathophysiology, wherefore electrical resistance was measured to assess changes in endothelial barrier function in real-time (Fig. 3b). The cells were cultured under low serum-containing conditions (1% FCS) and both untreated controls and PAH MVECs maintained an intact barrier over 72 h. In agreement with our staining and previous reports [14], BMP9 treatment stabilized the barrier in control cells that exhibited significantly higher resistance values at 72 h after stimulation compared to unstimulated samples (1629 ± 32 *vs*. 1961 ± 120 Ω, *p* = 0.001). On the opposite site, BMP9 caused a significant drop in PAH MVECs resistance after 24 h (2212 ± 92 *vs*. 1844 ± 105 Ω, *p* = 0.004) that continued to regress until a complete loss of barrier integrity at 72 h (1904 ± 160 *vs*. 269 ± 3 Ω, *p* < 0.001). Detailed analysis of the electrical parameters confirmed that BMP9 improved strength of cell–cell interactions significantly after 72 h (4.73 ± 0.16 *vs*. 6.36 ± 0.72, *p* = 0.004) in control cells, without affecting cell–matrix adhesions. BMP9 caused opposite effects in PAH MVECs and triggered an immediate opening of cell–cell contacts after stimulation that led to a complete loss of integrity at 72 h.

### Increased IL6 levels predispose PAH MVECs to BMP9-induced EndMT

**Fig. 4:**
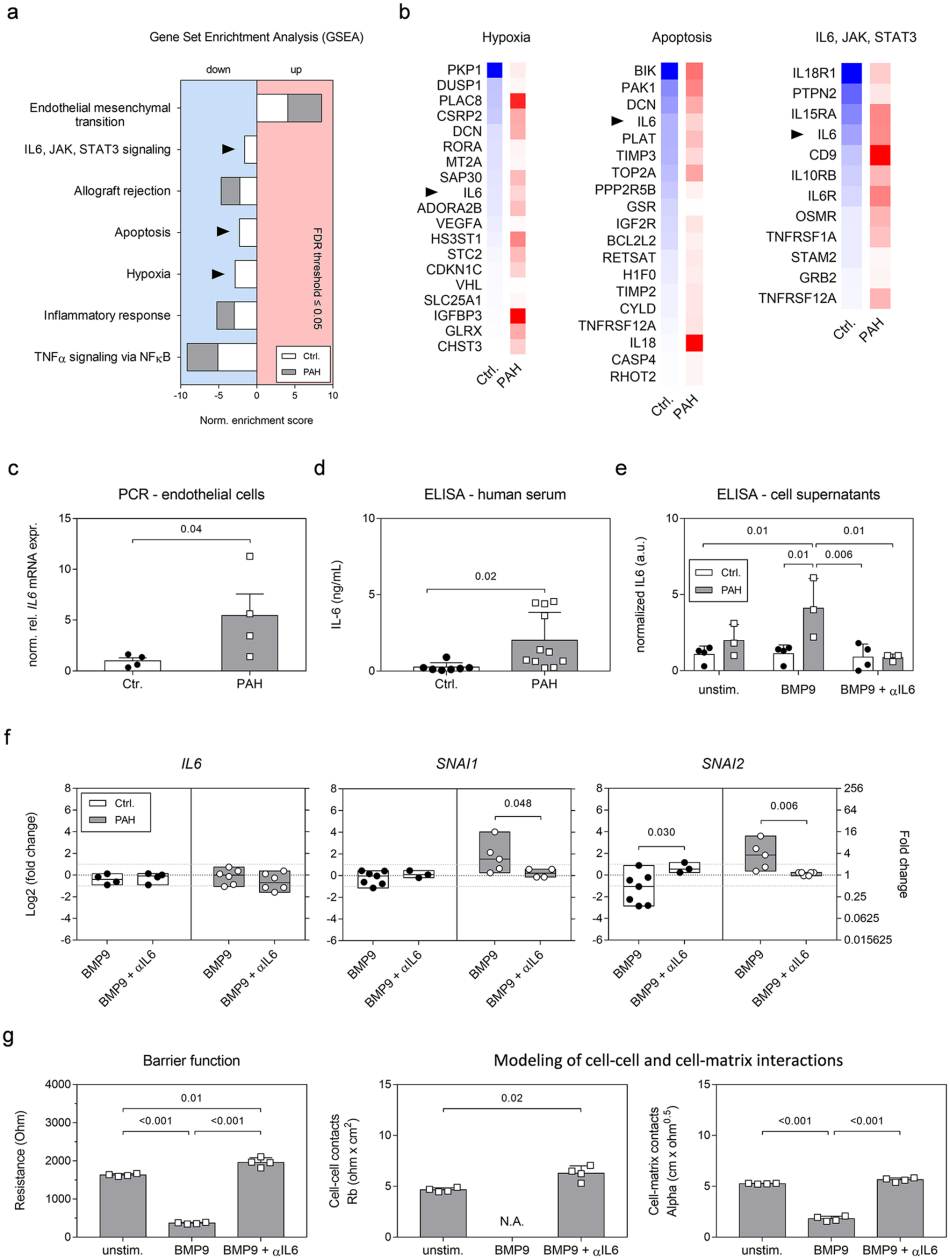
BMP9-induced persistent pro-inflammatory, pro-apoptotic, and pro-hypoxic signaling in the PAH endothelium is marked by increased IL6 levels. **a** Enrichment of PH signature pathways in control and PAH patient-derived MVECs based on the GSEA analysis. Arrowheads point out pathways in PAH samples that did not pass the enrichment threshold of FDR ≤ 0.05 and therefore are not regulated at 24 h after BMP9 treatment compared to unstimulated samples. **b** Switch gene analysis per non-enriched pathway and per donor group based on RNA-seq. Pseudocolors represent log2-fold decrease (blue) or increase (red) compared to non-stimulated samples. Arrow heads highlight interleukin-6 (IL6) as a common denominator between pathways. **c** Relative normalized IL6 mRNA expression in unstimulated human MVECs. **d** Total IL6 protein levels in human serum. **e** IL6 protein concentrations in cell-free culture supernatants of confluent human lung MVECs 24 h after different combination treatments with BMP9 and an IL6-capturing antibody (αIL6). **f** RT-PCR after 24-h treatment with BMP9 or BMP9 plus αIL6. Box plots represent min, max, and median log2-fold change compared to unstimulated conditions. Statistics were either calculated applying unpaired Student’s *t* tests (c, d, and f) or a two-way ANOVA with Tukey correction (**e**). **g** Quantification of barrier function and integrity of cell–cell and cell–matrix contacts (*n* = 4) by impedance spectroscopy at the 72-h time point. N.A. indicates inability to model data. Statistical differences were calculated by one-way ANOVA with Tukey correction

Our experiments revealed that BMP9 application induced an EndMT gene signature in both control and PAH samples on the short term, but only in PAH cells caused a mesenchymal phenotype on the long term. This is suggestive of a “second hit” needed to drive the PAH MVECs to a higher level of trans-differentiation. A detailed analysis of the seven disease-specific pathways, identified by GSEA, revealed missing negative enrichment (downregulation) of hypoxia; apoptosis; and IL6, JAK, STAT3 signaling in PAH samples (Fig. 4a). Associated with the missing negative enrichment were several genes that got induced in PAH pulmonary ECs and thereby inversely regulated compared to controls, so-called switch genes (Fig. 4b). Leading-edge analysis of all negatively enriched pathways identified *IL6* as the common denominator represented in six of the seven PH-associated pathways.

PCR analysis corroborated that *IL6* gene levels were five-fold increased under basal conditions in PAH cells (Fig. 4c). In accordance, ELISA measurements (Fig. 4d) detected significantly elevated IL6 levels in PAH patient serum. In vitro (Fig. 4e), a significant four-fold increased IL6 concentration compared to controls (1.1 ± 0.56 *vs*. 4.1 ± 1.95, *p* = 0.01) was measured in the cell-free supernatants of PAH MVECs after 24-h BMP9 stimulation. The increased IL6 levels were normalized to levels of controls by combining an IL6-capturing antibody (αIL6) with BMP9.

RT-PCR validated our findings that BMP9 did not directly induce transcription of *IL6* (Fig. 4f) but caused activation of *SNAI1* and *SNAI2* in PAH pulmonary ECs compared to untreated samples. Combination treatments with the αIL6 antibody prevented sustained induction of *SNAI1* (2.09 ± 1.43 *vs*. 0.23 ± 0.42, *p* = 0.048) and *SNAI2* (1.80 ± 1.31 *vs*. 0.13 ± 0.15, *p* = 0.006) and restored signaling to levels of unstimulated controls.

Functionally, normalization of autocrine IL6 levels by αIL6 impeded the BMP9-induced drop in electrical resistance (Fig. 4g). Blocking IL6 significantly improved PAH MVEC barrier functions (1629 ± 32.46 *vs*. 1961 ± 120.10 Ω, *p* = 0.01) and cell–cell contacts (4.65 ± 0.20 *vs*. 6.28 ± 0.73, *p *= 0.02) after 72-h BMP9 treatment while cell–matrix adhesion strength was maintained at levels of unstimulated conditions (5.26 ± 0.04 *vs*. 5.64 ± 0.19). In conclusion, our results suggest a central role for IL6-dependent signaling in the BMP9-induced EndoMT.

### Neutralization of autocrine IL6 prevents BMP9-induced EndMT of PAH pulmonary ECs

**Fig. 5:**
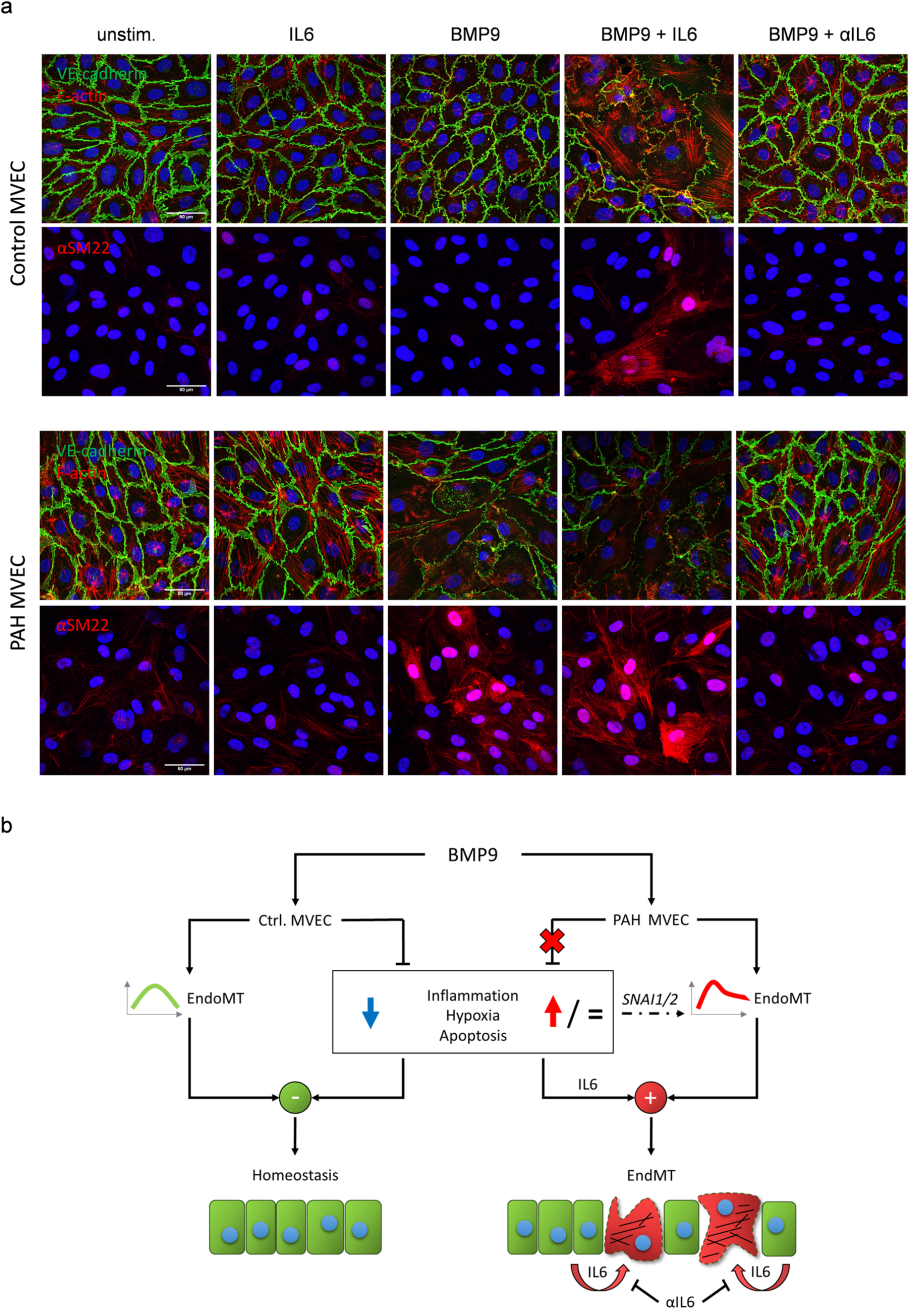
BMP9-induced phenotypic transformation of the PAH endothelium is mediated through IL6-dependent signaling and can be therapeutically impeded. **a** Representative immunostaining in control and PAH MVECs after combination treatments. Stimuli were applied every 24 h for three days total. **b** Explanatory model. BMP9 transiently activates EndMT transcriptional signaling in MVECs. In the healthy lung ECs this response is short-lived and on the long term coincides with a downregulation of other pathways including inflammatory, hypoxic, and apoptotic signaling. In MVECs of patients with PAH the suppression of pro-inflammatory, pro-hypoxic, and pro-apoptotic signaling upon BMP9 is astray. This causes persistent EndMT signaling in PAH marked by increased levels of the EndMT master transcription factors *SNAI1* and *SNAI2*. The loss of suppressor function is characterized by increased transcriptional levels of *IL6* as well as high levels of secreted IL6. Autocrine activation of the PAH endothelium by IL6 in conjunction with the BMP9-induced EndMT program causes the diseased lung ECs to lose endothelial-specific markers, gain mesenchymal characteristics, and decrease barrier integrity. The BMP9-triggered and IL6-mediated mesenchymal change and consequent loss of barrier function can be prevented by neutralizing endothelial secreted IL6 with a capturing antibody (αIL6)

We functionally tested the integrative role of IL6 as the modulator required to enable the mesenchymal change of pulmonary ECs. Confluent MVECs were treated by daily addition of BMP9, IL6, αIL6, a combination thereof, or left untreated for three days (Fig. 5a). Control cells receiving the combination of BMP9 plus IL6 lost peripheral VE-cadherin and gained organized SM22α protein expression, whereas single BMP9 treatment alone, in-line with previous experiments, induced a more closed or quiescent confirmation of cell–cell junctions with VE-cadherin tightly organizing at the junctions. In clear contrast, single BMP9 treatment as well as the combination of BMP9 and IL6 resulted in EndMT in PAH cells evident by the previously described changes in marker expression. Conversely, exposing the PAH MVECs to BMP9 in the presence of the αIL6 neutralizing antibody preserved endothelial-specific cobble stone morphology and cytoskeletal arrangement with sustained expression and junctional organization of VE-cadherin. These findings taken together with the PCR and ELISA data imply an autocrine mechanism for IL6 that can be therapeutically targeted.

## Discussion

In this study, we demonstrate that PAH microvascular lung ECs exhibited a significantly higher induction of *BMPR2* expression and the BMP target gene *ID1* upon stimulation with BMP9 than control pulmonary ECs and a distinctly different activation pattern than found in ECFCs from patients and controls. This suggests that tissue microenvironment and spatial differences/dysfunctions in the lung may control activation and outcome of BMP-dependent signaling in PAH. The distinct responses of PAH pulmonary ECs in comparison to controls were not due to differential BMPR2 nor downstream SMAD activation, which may indicate that alternative adaptive mechanisms fine-tune the response of microvascular ECs to BMP ligands in the lungs. We approached this hypothesis performing unbiased transcriptome analysis in control and PAH MVECs stimulated with BMP9. Our study revealed that most genes were similarly regulated by BMP9 in control and PAH cells at early time points, but after 24 h this regulatory pattern was lost in PAH cells pointing towards altered homeostatic or negative feedback signaling in PAH. Here, we identified a persistent enrichment in genes previously associated with EndMT and epithelial-to-mesenchymal transition (EMT) in the PAH lung ECs, including the EndMT master regulators *SNAI1* and *SNAI2* that are activated early in the EndMT process [7]. Accordingly, BMP9 effectively decreased the concentration of the EC marker VE-cadherin at cell–cell junctions while inducing the expression of transgelin (SM22α) in PAH cells leading to compromised endothelial barrier function, while in controls BMP9 even stabilized the barrier. Our pathway analysis identified IL6-dependent signaling to be an underlying mechanism that primes the PAH microvascular pulmonary ECs and enables EndMT upon BMP9 stimulation. Finally, using an IL6-capturing antibody, we demonstrated that the induction of EndMT and consequent loss of barrier function in PAH pulmonary MVECs is mediated by IL6 and triggered by BMP9 (Fig. 5b). Interestingly, we demonstrated that the combined action of IL6 and BMP9 may be a common and robust mechanism in cells from PAH donors representing the two most typical patient subtypes i) younger patients (before their 50s) with more severe hemodynamic impairments but better survival, and ii) an older subtype with more comorbidities [31].

EndMT is a highly integrative process that can result from tissue-specific pathway crosstalk induced by TGFβ family members, Notch and Wnt ligands, mechanical forces, growth factors, hypoxia, and inflammation [8]. The relative importance and order of activation depend on stimulus and/or underlying (patho)biology. The pro-inflammatory cytokine IL6 was previously shown to contribute to PH by commanding a proliferative and apoptosis-resistant pulmonary vasculature phenotype, to decrease BMPR2 levels, to exaggerate effects of chronic hypoxia, and to worsen vascular remodeling in BMPR2-deficient animals [32, 33]. Increased human serum IL6 protein levels are found across the systemic and lung circulation of PAH patients with mild-to-severe disease and are correlated with clinical phenotypes and outcomes in PAH subgroups [34, 35]. Additionally, we have shown that pro- and anti-inflammatory cytokine responses in MVECs of PAH patients are impaired [36]. In this study, however, we demonstrate that BMP9 or IL6 alone are not sufficient to induce full mesenchymal trans-differentiation, as previously suggested [37–39], wherefore we postulate that IL6 plays a mechanistic respectively modulating role. In accordance, we recently discovered that TNFα and IL‐1β induce EndMT in human primary aortic ECs by downregulation of BMPR2 which causes an altered signaling response to BMP9 and thereby sensitizing the cells for BMP9-induced osteogenic differentiation [40]. Similarly, BMP9 alone was reported to have no effect on monocyte and neutrophil recruitment to the vascular endothelium but amplifies the effects of pro-inflammatory stimuli like TNFα and LPS by priming the EC response [39, 41]. We and others thereby collectively hypothesize that imbalanced inflammatory signaling reactivates developmental programs that—in the diseased *milieu* of a PAH patient lung—continuously switches the EC phenotype between different pre-cursor states [42]. These transitional cells can easily be tipped towards one cell fate or another in response to injury or other triggers, as in this case BMP9.

Postnatally, EMT and EndMT are involved in the general lung repair program [43] but EndMT is also implicated in numerous pathogenesis including fibrotic diseases, cancer, atherosclerosis, and heterotopic ossification [8, 30, 44]. In PAH, EndMT was shown to potentially give rise to transitional cells co-expressing endothelial and mesenchymal markers that are found in up to 5% of the diseased lungs and abundantly within the typical vascular lesions [6, 37]. These transitional cells exert high proliferation rates with a migratory or even invasive phenotype that weakens the EC barrier [4]. However, due to the absence of EndMT-specific inhibitors it remains to be seen whether EndMT mediates the progression of PAH and aforementioned disorders or reflects an aberrant response to pathological stimuli in an attempt to initiate vascular repair and restore physiological function.

In conclusion, we provide evidence that BMP9-triggered EndMT signaling in conjunction with sustained pro-inflammatory, pro-hypoxic, and pro-apoptotic signaling mediated through IL6 causes an aberrant phenotypic trans-differentiation of the lung microvascular endothelium in PAH. Interestingly, despite all differences in age, gender, genetic background, and hemodynamics all PAH donor MVECs show similar responses to BMP9 going into EndMT in an IL6-dependent manner. Hence, we identify IL6 as a common factor modulating responses to BMP9 in end-stage PAH irrespective of the subtype. Accordingly, our study suggests that further investigations for the therapeutic use of BMP agonists in PAH should be pursued with attention to the features of EndMT as a possible indicator of long-term impact. Given the current findings, co-administration of anti-inflammatory therapy, such as an IL6 neutralizing antibody, could potentially mitigate inadvertent side effects of experimental drug candidates and might be considered as an add-on treatment for all subgroups of patients exerting high IL6 serum levels.

## Data Availability

Software for the Gene Set Enrichment Analysis (GSEA) can be downloaded from https://www.gsea-msigdb.org/gsea/index.jsp. Visualization of the GSEA was done with Cytoscape https://cytoscape.org/.

## Data availability

The datasets generated and/or analyzed during the current study are available from the corresponding author on reasonable request.

## References

[1] Simonneau G, Montani D, Celermajer DS, Denton CP, Gatzoulis MA, Krowka M, Williams PG, Souza R (2019). Haemodynamic definitions and updated clinical classification of pulmonary hypertension. Eur Respir J.

[2] Galiè N, Humbert M, Vachiery J-L, Gibbs S, Lang I, Torbicki A, Simonneau G, Peacock A, Vonk Noordegraaf A, Beghetti M, Ghofrani A, Gómez-Sánchez MA, Hansmann G, Klepetko W, Lancellotti P, Matucci M, McDonagh T, Pierard LA, Trindade PT, Zompatori M, Hoeper M (2016). 2015 ESC/ERS Guidelines for the diagnosis and treatment of pulmonary hypertension. The Joint Task Force for the Diagnosis and Treatment of Pulmonary Hypertension of the European Society of Cardiology (ESC) and the European Respiratory Society (ERS): Endorsed by: Association for European Paediatric and Congenital Cardiology (AEPC), International Society for Heart and Lung Transplantation (ISHLT). Eur Heart J.

[3] Rabinovitch M (2012). Molecular pathogenesis of pulmonary arterial hypertension. J Clin Invest.

[4] Suzuki T, Carrier EJ, Talati MH, Rathinasabapathy A, Chen X, Nishimura R, Tada Y, Tatsumi K, West JD (2018). Isolation and characterization of endothelial-to-mesenchymal transition cells in pulmonary arterial hypertension. Am J Physiol Lung Cell Mol Physiol.

[5] Stenmark KR, Frid M, Perros F (2016). Endothelial-to-mesenchymal transition. An evolving paradigm and a promising therapeutic target in PAH. Circulation.

[6] Ranchoux B, Antigny F, Rucker-Martin C, Hautefort A, Péchoux C, Bogaard HJ, Dorfmüller P, Remy S, Lecerf F, Planté S, Chat S, Fadel E, Houssaini A, Anegon I, Adnot S, Simonneau G, Humbert M, Cohen-Kaminsky S, Perros F (2015). Endothelial-to-mesenchymal transition in pulmonary hypertension. Circulation.

[7] Lamouille S, Xu J, Derynck R (2014). Molecular mechanisms of epithelial-mesenchymal transition. Nat Rev Mol Cell Biol.

[8] Sánchez-Duffhues G, García de Vinuesa A, ten Dijke P (2018). Endothelial-to-mesenchymal transition in cardiovascular diseases. Developmental signaling pathways gone awry. Dev Dyn.

[9] Rol N, Kurakula KB, Happé C, Bogaard HJ, Goumans M-J (2018). TGF-β and BMPR2 signaling in PAH. Two black sheep in one family. IJMS.

[10] Ogo T, Chowdhury HM, Yang J, Long L, Li X, Torres Cleuren YN, Morrell NW, Schermuly RT, Trembath RC, Nasim MT (2013). Inhibition of overactive transforming growth factor-β signaling by prostacyclin analogs in pulmonary arterial hypertension. Am J Respir Cell Mol Biol.

[11] Morrell NW, Bloch DB, ten Dijke P, Goumans M-JTH, Hata A, Smith J, Yu PB, Bloch KD (2016). Targeting BMP signalling in cardiovascular disease and anaemia. Nat Rev Cardiol.

[12] Spiekerkoetter E, Tian X, Cai J, Hopper RK, Sudheendra D, Li CG, El-Bizri N, Sawada H, Haghighat R, Chan R, Haghighat L, de Jesus Perez V, Wang L, Reddy S, Zhao M, Bernstein D, Solow-Cordero DE, Beachy PA, Wandless TJ, ten Dijke P, Rabinovitch M (2013). FK506 activates BMPR2, rescues endothelial dysfunction, and reverses pulmonary hypertension. J Clin Invest.

[13] Peiffer BJ, Qi L, Ahmadi AR, Wang Y, Guo Z, Peng H, Sun Z, Liu JO (2019). Activation of BMP signaling by FKBP12 ligands synergizes with inhibition of CXCR4 to accelerate wound healing. Cell Chem Biol.

[14] Long L, Ormiston ML, Yang X, Southwood M, Gräf S, Machado RD, Mueller M, Kinzel B, Yung LM, Wilkinson JM, Moore SD, Drake KM, Aldred MA, Yu PB, Upton PD, Morrell NW (2015). Selective enhancement of endothelial BMPR-II with BMP9 reverses pulmonary arterial hypertension. Nat Med.

[15] Urist MR (1965). Bone. Formation by autoinduction. Science.

[16] Goumans M-JTH, Zwijsen A, ten Dijke P, Bailly S (2018). Bone morphogenetic proteins in vascular homeostasis and disease. Cold Spring Harb Perspect Biol.

[17] Scharpfenecker M, van Dinther M, Liu Z, van Bezooijen RL, Zhao Q, Pukac L, Löwik CWGM, ten Dijke P (2007). BMP-9 signals via ALK1 and inhibits bFGF-induced endothelial cell proliferation and VEGF-stimulated angiogenesis. J Cell Sci.

[18] David L, Mallet C, Keramidas M, Lamandé N, Gasc J-M, Dupuis-Girod S, Plauchu H, Feige J-J, Bailly S (2008). Bone morphogenetic protein-9 is a circulating vascular quiescence factor. Circ Res.

[19] Levet S, Ouarne M, Ciais D, Coutton C, Subileau M, Mallet C, Ricard N, Bidart M, Debillon T, Faravelli F, Rooryck C, Feige J-J, Tillet E, Bailly S (2015). BMP9 and BMP10 are necessary for proper closure of the ductus arteriosus. Proc Natl Acad Sci USA.

[20] Brand V, Lehmann C, Umkehrer C, Bissinger S, Thier M, de Wouters M, Raemsch R, Jucknischke U, Haas A, Breuer S, Birzele F (2016). Impact of selective anti-BMP9 treatment on tumor cells and tumor angiogenesis. Mol Oncol.

[21] García de Vinuesa A, Abdelilah-Seyfried S, Knaus P, Zwijsen A, Bailly S (2016). BMP signaling in vascular biology and dysfunction. Cytokine Growth Factor Rev.

[22] Tu L, Desroches-Castan A, Mallet C, Guyon L, Cumont A, Phan C, Robert F, Thuillet R, Bordenave J, Sekine A, Huertas A, Ritvos O, Savale L, Feige J-J, Humbert M, Bailly S, Guignabert C (2019). Selective BMP-9 inhibition partially protects against experimental pulmonary hypertension. Circ Res.

[23] Wang G, Fan R, Ji R, Zou W, Penny DJ, Varghese NP, Fan Y (2016). Novel homozygous BMP9 nonsense mutation causes pulmonary arterial hypertension. A case report. BMC Pulm Med.

[24] Hiepen C, Jatzlau J, Hildebrandt S, Kampfrath B, Goktas M, Murgai A, Cuellar Camacho JL, Haag R, Ruppert C, Sengle G, Cavalcanti-Adam EA, Blank KG, Knaus P, Mullins MC (2019). BMPR2 acts as a gatekeeper to protect endothelial cells from increased TGFβ responses and altered cell mechanics. PLoS Biol.

[25] Szulcek R, Happé CM, Rol N, Fontijn RD, Dickhoff C, Hartemink KJ, Grünberg K, Tu L, Timens W, Nossent GD, Paul MA, Leyen TA, Horrevoets AJ, de Man FS, Guignabert C, Yu PB, Vonk-Noordegraaf A, van Nieuw Amerongen GP, Bogaard HJ (2016). Delayed microvascular shear adaptation in pulmonary arterial hypertension role of platelet endothelial cell adhesion molecule-1 cleavage. Am J Respir Crit Care Med.

[26] Smits J, Tasev D, Andersen S, Szulcek R, Botros L, Ringgaard S, Andersen A, Vonk-Noordegraaf A, Koolwijk P, Bogaard HJ (2018). Blood Outgrowth and proliferation of endothelial colony forming cells are related to markers of disease severity in patients with pulmonary arterial hypertension. IJMS.

[27] Szulcek R, Bogaard HJ, van Nieuw Amerongen GP (2014). Electric cell-substrate impedance sensing for the quantification of endothelial proliferation, barrier function, and motility. J Vis Exp.

[28] Subramanian A, Tamayo P, Mootha VK, Mukherjee S, Ebert BL, Gillette MA, Paulovich A, Pomeroy SL, Golub TR, Lander ES, Mesirov JP (2005). Gene set enrichment analysis: a knowledge-based approach for interpreting genome-wide expression profiles. Proc Natl Acad Sci USA.

[29] Szklarczyk D, Franceschini A, Wyder S, Forslund K, Heller D, Huerta-Cepas J, Simonovic M, Roth A, Santos A, Tsafou KP, Kuhn M, Bork P, Jensen LJ, von Mering C (2015). STRING v10. Protein-protein interaction networks, integrated over the tree of life. Nucleic Acids Res.

[30] Evrard SM, Lecce L, Michelis KC, Nomura-Kitabayashi A, Pandey G, Purushothaman K-R, d’Escamard V, Li JR, Hadri L, Fujitani K, Moreno PR, Benard L, Rimmele P, Cohain A, Mecham B, Randolph GJ, Nabel EG, Hajjar R, Fuster V, Boehm M, Kovacic JC (2016). Endothelial to mesenchymal transition is common in atherosclerotic lesions and is associated with plaque instability. Nat Commun.

[31] Ling Y, Johnson MK, Kiely DG, Condliffe R, Elliot CA, Gibbs JSR, Howard LS, Pepke-Zaba J, Sheares KKK, Corris PA, Fisher AJ, Lordan JL, Gaine S, Coghlan JG, Wort SJ, Gatzoulis MA, Peacock AJ (2012). Changing demographics, epidemiology, and survival of incident pulmonary arterial hypertension. Results from the pulmonary hypertension registry of the United Kingdom and Ireland. Am J Respir Crit Care Med.

[32] Pickworth J, Rothman A, Iremonger J, Casbolt H, Hopkinson K, Hickey PM, Gladson S, Shay S, Morrell NW, Francis SE, West JD, Lawrie A (2017). Differential IL-1 signaling induced by BMPR2 deficiency drives pulmonary vascular remodeling. Pulm Circ.

[33] Tamura Y, Phan C, Tu L, Le Hiress M, Thuillet R, Jutant E-M, Fadel E, Savale L, Huertas A, Humbert M, Guignabert C (2018). Ectopic upregulation of membrane-bound IL6R drives vascular remodeling in pulmonary arterial hypertension. J Clin Invest.

[34] Selimovic N, Bergh C-H, Andersson B, Sakiniene E, Carlsten H, Rundqvist B (2009). Growth factors and interleukin-6 across the lung circulation in pulmonary hypertension. Eur Respir J.

[35] Simpson CE, Chen JY, Damico RL, Hassoun PM, Martin LJ, Yang J, Nies M, Griffiths M, Vaidya RD, Brandal S, Pauciulo MW, Lutz KA, Coleman AW, Austin ED, Ivy DD, Nichols WC, Everett AD (2020). Cellular sources of interleukin-6 and associations with clinical phenotypes and outcomes in pulmonary arterial hypertension. Why novel is not always best. Eur Respir J.

[36] Dummer A, Rol N, Szulcek R, Kurakula K, Pan X, Visser BI, Bogaard HJ, DeRuiter MC, Goumans M-J, Hierck BP (2018). Endothelial dysfunction in pulmonary arterial hypertension. Loss of cilia length regulation upon cytokine stimulation. Pulm Circ.

[37] Good RBB, Gilbane AJJ, Trinder SLL, Denton CPP, Coghlan G, Abraham DJJ, Holmes AMM (2015). Endothelial to mesenchymal transition contributes to endothelial dysfunction in pulmonary arterial hypertension. Am J Pathol.

[38] Hopper RK, Moonen J-RAJ, Diebold I, Cao A, Rhodes CJ, Tojais NF, Hennigs JK, Gu M, Wang L, Rabinovitch M (2016). In pulmonary arterial hypertension, reduced BMPR2 promotes endothelial-to-mesenchymal transition via HMGA1 and its target slug. Circulation.

[39] Appleby SL, Mitrofan C-G, Crosby A, Hoenderdos K, Lodge K, Upton PD, Yates CM, Nash GB, Chilvers ER, Morrell NW (2016). Bone morphogenetic protein 9 enhances lipopolysaccharide-induced leukocyte recruitment to the vascular endothelium. J Immunol.

[40] Sánchez-Duffhues G, García de vinuesa A, van Pol V, Geerts ME, de Vries MR, Janson SG, van Dam H, Lindeman JH, Goumans M-JTH, ten Dijke P (2019). Inflammation induces endothelial-to-mesenchymal transition and promotes vascular calcification through downregulation of BMPR2. J Pathol.

[41] Mitrofan C-G, Appleby SL, Nash GB, Mallat Z, Chilvers ER, Upton PD, Morrell NW (2017). Bone morphogenetic protein 9 (BMP9) and BMP10 enhance tumor necrosis factor-α-induced monocyte recruitment to the vascular endothelium mainly via activin receptor-like kinase 2. J Biol Chem.

[42] Cooley BC, Nevado J, Mellad J, Yang D, Hilaire CS, Negro A, Fang F, Chen G, San H, Walts AD, Schwartzbeck RL, Taylor B, Lanzer JD, Wragg A, Elagha A, Beltran LE, Berry C, Feil R, Virmani R, Ladich E, Kovacic JC, Boehm M (2014). TGF-β signaling mediates endothelial-to-mesenchymal transition (EndMT) during vein graft remodeling. Sci Transl Med.

[43] Chapman HA (2012). Epithelial responses to lung injury. Role of the extracellular matrix. Proc Am Thorac Soc.

[44] Lin F, Wang N, Zhang T-C (2012). The role of endothelial-mesenchymal transition in development and pathological process. IUBMB Life.

